# The ‘big four’ health risk behaviors among Vietnamese adolescents: co-occurrence and socio-cultural risk factors

**DOI:** 10.1080/21642850.2022.2057314

**Published:** 2022-04-05

**Authors:** Hoang-Minh Dang, Ha Ho, Bahr Weiss

**Affiliations:** Center of Research, Information and Services in Psychology, VNU University of Education, Cau Giay, Hanoi, Vietnam

**Keywords:** Health risk behaviors, tobacco and alcohol use, physical inactivity, unhealthy diet, adolescence, low-and-middle income countries, Vietnam

## Abstract

**Background:**

Health Risk Behaviors (HRBs) represent significant health threats for adolescents. However, there has been relatively little research on multiple HRBs in low-and-middle-income countries (LMIC), where the majority of the world’s youth reside. This study’s objective was to investigate common HRB, their co-occurrence, and socio-demographic risk and protective factors among Vietnamese adolescents.

**Methods:**

A cross-sectional self-report survey examined four major HRBs (tobacco use, alcohol consumption, physical inactivity, unhealthy diet) among 431 adolescents aged 15–17 years in five Vietnam provinces. Key HRB risk and protective factors assessed included perceived social norms, parental monitoring of adolescents’ behavior, and health behavior literacy.

**Results:**

Forty-one percent of participants reported no HRB, 39% reported one, and 20% reported two or more HRBs. The four HRBs appeared to be independent, with an exploratory factor analysis indicating no common factors. The most prevalent HRB was unhealthy diet (45%), the least prevalent smoking (1%). The most frequent co-occurring HRBs were unhealthy diet and physical inactivity. Adolescents’ perceptions of norms regarding HRBs and related healthy behaviors were the most consistent risk factor for the HRB. Diet was the HRB most influenced by the social variables perceived norms, monitoring, and health literacy.

**Conclusions:**

Results highlight the importance of future research identifying the temporal order of co-occurrence of multiple HRB, how differing conceptualizations of socio-cultural roles impact on HRB, and health-related effects of HRB co-occurrence. Such information will be useful for maximizing the efficiency and effectiveness of prevention and intervention programs in LMIC.

## Introduction

‘Health risk behaviors’ (HRBs) are behaviors that directly or indirectly influence physical health and wellbeing (DiClemente, Hansen, & Ponton, 2013). HRBs can be grouped into two major categories: (a) behaviors that are harmful (e.g. smoking; drinking alcohol); (b) behaviors where a failure to engage in the behavior is harmful (e.g. low physical activity) (DiClemente, Santelli, & Crosby, 2009). There are a number of such HRBs, but public health often has focused on four of the most prevalent and impactful, sometimes called the ‘Big Four’ HRBs (World Health Organization, 2003) – tobacco use, harmful use of alcohol, physical inactivity, unhealthy diet – because they are major risk factors for chronic non-communicable diseases and contribute globally to about fifty percent of premature deaths (World Health Orgniazation, 2021). For instance unhealthy diet, characterized by excessive saturated fat, sugar, salt, etc., is associated with many chronic and severe diseases such as Type II diabetes, cancer and cardiovascular diseases (Te Morenga & Montez, 2017). Tobacco use is the leading preventable cause of death, with 6.4 million deaths per year worldwide (Reitsma et al., 2017).

Although many consequences of such behaviors occur in mid-life, adolescence is a critical time-period because it is when the behaviors often begin and become habitual (Djoussé, Driver, & Gaziano, 2009), increasing long-term risk for disease and pre-mature mortality. Late adolescence is a particularly critical time period because it is immediately prior to when legal adulthood begins in many countries, which can result in reduced parental influence regarding health-related behaviors (Weinstein, 2004). These HRBs among adolescents also can impose significant economic and social burdens on society (Kann et al., 2018). According to a study conducted by the World Health Organization in 50 countries (including LMIC) from the early 1980s to 2020, a significantly increasing number of adolescents engage in HRBs (i.e. smoking, alcohol drinking, physical inactivity, sexual behavior) (Currie, Gabhainn, & Godeau, 2009; Incheley et al., 2020). In the U.S., 66% of adolescents aged 12–18 reported at least one significant HRB (Heneghan et al., 2015). In less developed countries, rates are similar or higher. In Brazil, for instance, it was found that 98%  of the high school students had at least one HRB, with almost 10% engaging in four behaviors (Brito, Hardman, & Barros, 2015).

In fact, there is strong evidence that adolescents tend to engage in multiple HRBs, not single HRB (Campbell et al., 2020) i.e. adolescents who engage in one type of health-risk behavior such as alcohol use may be more likely to engage in other types of HRB, such as smoking. Data from the USA, for instance, found that 33% of adolescents aged 14–17 years engage in two or more HRBs (Spring, Moller, & Coons, 2012). Dumith, Muniz, Tassitano, Hallal, & Menezes (2012) reported that over 60% of Brazilian adolescents had co-occurrence of two or more risk behaviors. This means that prevention and intervention programs will be most efficient if they can target the mechanisms underlying this co-occurrence, versus having separate programs for each HRB. The specific combinations of these HRBs varies in different populations (Spring et al., 2012), but the issue of efficiency is particularly important in LMIC with their limited resources, However, at present there is still debate regarding the mechanisms underlying these associations between HRB. Two main models have been posited. First, it has been hypothesized that there is a ‘gateway’ process, where a person engaging in one type of HRB leads to others through increased exposure and desire to engage in other unsafe behaviors, in part through a decreased sense of the perceived danger of other risk behavior, given the apparent lack of immediate consequences for the initial HRB (Hale & Viner, 2016). A second hypothesis is that comorbidity of HRB reflects a set of common psychological or social factors risk factor for HRB (Alamian & Paradis, 2012; Jackson, Henderson, Frank, & Haw, 2012). For example, Jessor (2016)’s ‘problem behavior theory’ suggests that multiple risk behaviors may occur as a function of a single behavioral syndrome reflecting a general dimension of unconventionality or rebelliousness. The likelihood of development of such a syndrome is based on psychosocial protective and risk factors related to emotional well-being, and connection to the conventional institutions of family, school and religion (Jessor & Turbin, 2016). According to this theory, the co-occurrence of HRBs in adolescence reflects the common goal of an adolescent demonstrating to his or her community independence and maturity, and repudiating conventionality (Jessor, Turbin, & Costa, 1998).

The present study focused on the Southeast Asian LMIC of Vietnam. HRBs are particularly important in LMICs because of relatively limited health system capacity and research capacity to identify and reduce risk, and to prevent or treat consequent health problems. Improving the health of adolescents in LMICs is considered essential for the world to achieve the United Nations Sustainable Development goals, and the specific targets and goals included in the United Nations Secretary General’s Global Strategy for Women’s, Children’s and Adolescents’ Health (UN General Assembly, 2015). Although there has been an increased call for research on the health and wellbeing of adolescents and young people to guide these and other global and national initiatives, research in LMICs has been and remains limited (Xu et al., 2020). In addition, because HRBs are highly embedded in culture and influenced by cultural norms (e.g. regarding rebelliousness), research in a range and variety of countries is critical. For instance, in Europe the gender ratio for smoking is close to 1 (i.e. equal numbers of males and females smoke) whereas in the Vietnam it is close to 10/1 (World Health Organization, 2015), strongly suggesting the influence of culture on the development. Adolescents’ eating behaviors, for instance, are strongly formed by social environment (e.g. family socioeconomic status, peers and friends’ norms; (Kabir, Miah, & Islam, 2018) and culture (Levine et al., 2016)). Thus, it is important to assess HRBs in a range of countries. The prevalence of HRBs in LMICs has been estimated in several studies, including in Vietnam (Pengpid et al., 2016). However, research in Vietnam is relatively old (Nguyen, Rahman, Emerson, Nguyen, & Zabin, 2012; Phuong, Huong, Tien, Chi, & Dunne, 2013), which given the strong link between HRB and social factors which have been rapidly changing in countries like Vietnam, is a critical limitation. The extant research in Vietnam is also geographically limited (Phuong et al., 2013), and/or focused on a single HRB (Nguyen, Hong, Hoang, Nguyen, & Robert, 2013; Nguyen, Tang, Dibley, & Sibbritt, 2009). Finally, to the best of our knowledge, no studies have been conducted investigating multiple HRBs among Vietnamese adolescents. The goals of the present study therefore were to estimate the prevalence of single and co-concurrent HRBs (unhealthy dietary behaviors, physical inactivity, tobacco and alcohol consumption), and to explore HRBs’ associations with social-cultural factors among adolescents in five provinces in Vietnam.

## Methods

### Participant and sampling frame

The present study conducted a cross-sectional assessment of four HRBs (unhealthy dietary behaviors, physical inactivity, tobacco consumption, alcohol consumption) among Vietnamese adolescents aged 15–17 years, using stratified random sampling. The goal of the sampling frame was to sample a range of socio-economic conditions in Vietnam. Five provinces were thus purposively selected to provide varying (a) levels of urbanization, (b) socio-economic conditions, and (c) geographical character (coastal vs. inland; north vs center vs south). Provinces included Hanoi (the national capital, highly urbanized, northern and inland), Lao Cai (rural, north-western, mountain region), Ninh Binh (semi-rural, north-central, inland), Khanh Hoa (semi-rural, south-central, coastal), Dong Nai (urban, southern, inland). For data collection feasibility, only adolescents attending high schools were recruited. Adolescents were recruited through public schools, which the large majority of Vietnamese children attend (General Statistics Office of Vietnam, 2019). Within each province except Hanoi, three high-schools were purposively selected so as to best reflect the urban vs. rural nature of the province; because Hanoi is much larger than the other provinces, six schools were selected there, using the same guidelines. In each school, stratified random sampling was used to select 24 students from grade 10 and from grade 11th to recruit for study participation. Four students were randomly selected from each of three randomly selected classes within each grade. For the inferential analyses, the minimum sample size was set at 415 participants, in order to produce power of .80 to detect an effect size of δ =  .275 (Spybrook et al., 2011). The total number of students invited to participate in the study was 432. The number of students whose parents provided consent and the adolescent provided assent was 431, for a 99% participation rate. The one exclusion criterion was a lack of adolescent or parental consent. The mean age of participants was 15.6 (SD = 0.6). Sample description is provided in Table 1. STROBE guidelines for cross-sectional research (Vandenbroucke et al., 2007) were used in this study and manuscript.

**Table 1.** Sample characteristics.

Variable	Mean	STD
Age (range = 15 to 17)	15.64	0.59
	**N**	**%**
Gender		
Female	209	49
Male	222	51
Residence area		
Urban area	287	67
Non-urban area	144	33
Family structure		
Living with both parents	369	86
Parents separated or divorced	33	8
Parent deceased	28	6

Note: Total N = 431.

### Measures

*Demographic information:* Socioeconomic and demographic information including age, residential area (urban, vs. non-urban), gender (male/female) and other similar variables were collected in a demographic questionnaire.

*Physical inactivity:* Participants’ physical activity was assessed using the International Physical Activity Questionnaire (IPAQ Research Committee, 2005). The IPAQ covers four domains of physical activity: work-related, transportation, housework/gardening, and leisure-time activity. In each of the four domains the number of days per week and time per day spent in both moderate and vigorous activity are recorded. To assist participants in the recall (e.g. for time spent walking), they were given a 7-day monitoring form to track physical activities for one week prior to the study assessment. Scoring of the IPAQ followed the IPAQ *Guidelines* (IPAQ Research Committee, 2005). Continuous data are reported using the IPAQ scoring system that summarizes the intensity of physical activity, with an overall Total Score for physical activity based on for MET (metabolic equivalent) minutes per week score. In the present study, internal reliability for the Total Score scale was 0.74. Participants also were classified into high, moderate and low levels of activity, based on their Total Score (IPAQ Research Committee, 2005). For the purposes of this study, ‘physical inactivity’ was defined as being in the IPAQ ‘low’ level (Dumith, Hallal, Reis, & Kohl III, 2011).

*Dietary behaviors:* Participants’ dietary behaviors were evaluated using the Adolescent Food Habits Checklist (AFHC) (Johnson, Wardle, & Griffith, 2002), a measure of adolescent eating behavior widely used around the world (Kalkan, 2019). The AFHC consists of 23 items evaluating consumption of healthy foods (e.g. fruits and vegetables), unhealthy foods (e.g. fats, fast-food), and daily diet patterns in general. Participants answer ‘true’ or ‘false’ or ‘not applicable’ regarding whether they typically engage in the dietary practice. Total scores were calculated, adjusting the number of healthy responses for the number of ‘not applicable’ and missing responses (AFHC score = [number of ‘healthy’ responses – number of ‘unhealthy’ responses] × [23/number of items completed]); thus, a negative score indicates more unhealthy than healthy responses. The AHFC does not have specific guidelines for binary categorization into ‘healthy’ or ‘unhealthy’ eating, therefore for the present study, ‘unhealthy’ eating was defined as having more unhealthy eating behaviors than healthy eating behaviors. In this study, the Total Score had an internal consistency of 0.77.

*Tobacco and alcohol consumption*: Four items taken from U.S. Centers for Disease Control *National Youth Tobacco Survey* (US.CDC, 2015; Weiss, Nguyen, Trung, Ngo, & Lau, 2019) were used to assess tobacco smoking; parallel items similarly worded were used to assess alcohol use. These items assessed (a) lifetime smoking, or lifetime alcohol drinking, (b) when the most recent use was, (c) the number of times tobacco, or alcohol, was used in the past 30 days, and (d) the number of times tobacco, or alcohol, was typically used in a day. Items ‘c’ and ‘d’ were standardized within domain and summed to provide continuous smoking and drinking variables. Because only two items were used to compute each of these variables, internal consistency estimates were not computed. Binary variables for tobacco use and alcohol consumption were assessed as any use.

*Perceived norms for health risk behaviors:* Students’ perceived social norms for diet and physical activity were assessed with the Perceived Social Norms measure (Ball, Jeffery, Abbott, McNaughton, & Crawford, 2010). For each of 18 items across these two domains, students indicated their level of agreement on a Likert 5-point scale. One set of items assessed descriptive norms regarding parents’ and (separately) peers’ behavior (e.g. ‘*My friends often eat fast food’*); the second set of items assessed perceptions of injunctive norms, parents’ and peers’ approval of these behaviors (e.g. ‘‘*My parents think I should eat healthily’*)*.* For alcohol and tobacco use, descriptive norms were assessed by asking students to estimate the prevalence of smoking and drinking for adults and youth, with five responses options ranging from 0–20% to 81%-100%. To assess injunctive norms for alcohol and tobacco use, students rated the extent to which (a) their parents, (b) adults in general, and (c) their peers approved or disapproved of these behaviors, on a 0 = strongly disapprove to 4 = strongly approve scale. Internal consistency for all four of the scales was in the acceptable range, 0.68, 0.63, 0.68., and 0.71 for respectively Diet, Physical Activity, Alcohol, and Tobacco.

*Parental Monitoring:* The Parental Monitoring measure, based on the Stormshak, Caruthers, and Dishion (2006) measure, assessed adolescents’ beliefs about the extent to which their parents were aware of what the adolescent was doing during free time, outside the home, etc. (e.g. ‘*How often do your parents know what you do during your free time’*). Parents completed a similar questionnaire, worded in parallel. Responses are rated on a 0 = Never to 4 = Always scale with items summed, within informant, to produce a separate Parental Monitoring score, by informants. Internal consistency for the child-report of parental monitoring was 0.82 and for parent-report 0.83.

*Health behavior literacy:* Students and parents completed a health behavior literacy inventory, based on WHO guidelines regarding physical activity, diet, and alcohol and tobacco consumption (World Health Organization, 2003, 2015). The inventory assesses knowledge about the negative health consequences of HRB (e.g. ‘*An unhealthy diet can cause diabetes’*), rated on a 0 = strongly disagree to 4 = strongly agree scale. It also assessed knowledge regarding specific dangers associated with each of the HRB (e.g. ‘*Tobacco is an addictive substance’)*. Items are summed within informant to create a total health behavior literacy score, separately by informant. Internal consistency for child-report was 0.73 and for parent-report 0.76.

All measures were culturally adapted and translated into Vietnamese, and back translated by the research team which includes Vietnamese psychologists fluent in English, and an American psychologist fluent in Vietnamese, using standard procedures to maintain the semantic, technical, and conceptual content of the measure (e.g. Byrne, 2016; Hambleton, 2005). The research team is highly experienced in measure adaptation and translation, being the official translator and distributor for a number of well-respected assessment instruments (e.g. the WISC-IV;(Dang, Weiss, Pollack, & Nguyen, 2012); the Child Behavior Checklist; (Dang, Nguyen, & Weiss, 2017) in Vietnam. To confirm the readability of the measures, the measures were piloted with 10 high school students to ensure that the questions and answer options were clearly written and understood correctly.

### Data collection procedures

A letter introducing the project was sent to the provincial Department of Education and Training (DOET) in the five selected provinces. All DOET contacted were interested in and agreed to participate in the study. Project research assistants contacted principals of selected schools to discuss the project details. All schools agreed to participate in the project, after which research assistants randomly selected three classes per grade, then four students per class from a blinded student list. Students were contacted by their teacher and informed about the study. The study was discussed in detail with those interested; all students voluntarily provided assent and were given an introduction letter and consent form for their parents. All but one student’s parents gave permission for their child to participate.

After students’ assent and parental consent were obtained, a meeting with all participating students in each school was held during a free period, and the students were instructed how to complete a 7-day monitoring form of their physical activity and eating behavior. After returning the completed form one week later, researcher assistants administered the main survey during available times arranged by the school. Each participant received compensation of 50,000 VND (equivalent to about US $2.50). The study was approved by the Institutional Review Board of the University.

### Statistical analyses

Analyses were conducted using SAS (9.4). Means, frequencies and confidence intervals for the descriptive analyses were computed using SAS Proc Means and Proc Freq. As part of the descriptive analyses, to summarize relations among the four HRB, we conducted an exploratory factor analysis in SAS Proc Factor, using iterated principal factor analysis estimation, with squared multiple correlations as the initial communality estimates.

For the primary inferential analyses for the IPAQ and AFHC, general linear models (GLM) were used via SAS Proc GLM with the two HRB as the dependent variables in separate models. In the primary inferential analyses for Drinking and Smoking, ordinal models were used in logistic regression with a cumulative link function, in SAS Proc Logistic. The categorical independent variables included gender, residence, and family structure; continuous independent variables included the perceived norms, parental monitoring, and health literacy variables. Subscales for the independent variables (e.g. for Perceived Norms: healthy diet; physical activity; drinking; smoking) were included in the same model (e.g. in the model with Perceived Norms as the independent variable) to control for confounding across the subscales. These analyses were also run with Province included as a random categorical (df = 4) effect, but because inclusion of Province had minimal effects on results, for the sake of parsimony analyses without Province are reported below.

## Results

### Preliminary analyses

Prevalence of individual health risk behaviors are reported in Table 2. Across the four HRBs, the most prevalent HRB was unhealthy diet (45%), and the least prevalent smoking (1%). Of the 431 adolescents, 41% reported no HRB, 39% reported one HRB, 19% reported two HRBs, and 1% reported three HRBs; no participants reported involvement in all four HRBs (Table 3). The most frequent combination of two HRB was unhealthy diet and physical inactivity, which follows logically from the rates of the individual HRB (i.e. unhealthy diet and physical inactivity were the first and second most frequently reported HRB). Correlations of the four HRB were assessed and reported in Table 4. To summarize the magnitudes of these relations among the HRB, we computed the internal consistency for the four continuous scales. The internal consistency alpha was .07, indicating that the four scales were essentially independent. The results of the exploratory factor analysis on the four scales confirmed this result. The first eigenvalue from the matrix was less than 1.00 (0.22) indicating there were no common factors underlying these four scales, i.e. they represent four independent behaviors.

**Table 2.** Prevalence of individual health risk behaviors.

Health Risk Behavior	N (of total sample)	Percent (95% confidence interval)
Unhealthy Diet	194 (431)	45% (40% to 50%)
Low Physical Activity	96 (419)	23% (19% to 27%)
Drinking (alcohol)	57 (430)	13% (10% to 16%)
Smoking (tobacco)	4 (431)	1% (0% to 2%)

**Table 3.** Comorbidity of health risk behaviors.

# of HRB per participant	Health Risk Behavior(s)	# of participants, % (95% confidence interval)
**0**		**174 (of 431) 41%(36% to 45%)**
**1**		**169 (of 431) 39% (35% to 44%)**
	Diet	110 65% (58% to 72%)
	Physical	32 19% (13% to 25%
	Drinking	25 15% (9% to 20%)
	Smoking	2 1% (0% to 3%)
**2**		**82 (of 431) 19% (15% to 23%)**
	Diet, Physical	55 67% (57% to 77%)
	Diet, Drinking	22 27% (17% to 37%)
	Diet, Smoking	1 1% (0% to 4%)
	Physical, Drinking	4 5% (0% to 10%)
**3**		**6 (of 431) 1% (0% to 3%)**
	Diet, Physical, Drinking	5 83% (40% to 100%)
	Diet, Drinking, Smoking	1 17% (0% to 60%)
**4**		**0 (of 431) 0%**

**Table 4.** Correlations among continuous health risk behavior scales.

HRB	AFHC	IPAQ	Drinking	Smoking
AFHC	1.00			
IPAQ	0.15**	1.00		
Drinking	0.06	−.11*	1.00	
Smoking	0.04	0.03	−.01	1.00

Note: AFHC = Adolescent Food Habits Checklist; IPAQ = International Physical Activity Questionnaire. The AFHC and IPAQ questionnaires are reverse scored for this table, so that all four behaviors are scored in the same direction (higher scores indicating higher levels of unhealthy behaviors). * = *p* < .05, ** = *p* < .01.

### Primary analyses

The next analyses focused on socio-cultural risk factors for the HRBs, with the first factor assessed gender. Males and females did not differ significantly for HRB Diet or Drinking but did on for Physical Activity, with females approximately .35 of an SD lower than males. The model for Gender predicting Smoking was non-estimable, because all four of the participants who reported smoking were male. Non-urban (consisting of rural and semi-rural) and urban areas did not differ on any HRB, with effect on Smoking non-estimable, because all participants who reported smoking were from non-urban areas. Family structure (living with both parents, or not) differed significantly predicted Drinking, with participants who were not living with both parents having odds of reporting drinking almost three and a half times that for participants who reported living with both parents. Adolescents’ perceptions of health-related norms were analyzed in four models across the four HRB as the dependent variable, with each domain of norms (diet, physical activity, drinking, smoking) as the independent variables in all four models (see Table 5). The overall effect of the four norms was significant for Diet (AFHC), F(4,413) = 13.54, *p* < .0001, R^2 ^= 0.12, indicating that adolescents’ perceptions of health-related norms were related to their levels of Healthy Diet. Within this model, perceptions of norms regarding both diet and physical activity had significant effects, F(1,413) = 31.58, *p* < .0001, *β* = 0.28 and F(1,413) = 6.25, *p* < .05, *β* = 0.12 (respectively) on adolescent eating behavior. As would be expected, the beta effect size for norms regarding diet was larger than the beta for physical activity, by a factor of more than two: β = 0.28 and β = 0.12, respectively; i.e. norms regarding diet were more strongly related to adolescents’ reported dietary behaviors than norms regarding physical activity.

**Table 5.** Demographic and health-related predictors of the health risk behavior scales.

		DV			
	IV	AFHC	IPAQ	Drinking	Smoking
**Gender**^a^		**F(1,416) = 0.20**	F(1,416) = 12.88***, R^2 ^= 0.03, β = 0.17	**χ[1] = 1.66**	**Non-estimable**^b^
**Residence (urban vs. non-urban)**		**F(1,416) = 0.43**	**F(1,416) = 0.83**	**χ[1] = 0.00**	**Non-estimable**^b^
**Family structure**		**F(2,414) = 0.39**	**F(2,414) = 0.12**	χ[1] = 4.02*, OR = 3.40	**χ[1] = 0.39**
**Norms**^c^	**Total Model**	F(4,413) = 13.54****, R^2 ^= 0.12	F(4,413) = 9.04****, R^2 ^= 0.08	χ[4] = 24.75****, R^2 ^= 0.13	**χ[4] = 7.39**
	Healthy Diet	F(1,413) = 31.58****, β = 0.28	F(1,413) = 0.01	χ[1] = 0.00	
	Physical Activity	F(1,413) = 6.25*, β = 0.12	F(1,413) = 19.27****, β = 0.22	χ[1] = 1.70	
	Drinking	F(1,413) = 0.18	F(1,413) = 10.82**, β = 0.20	χ[1] = 24.25****, OR = 3.62	
	Smoking	F(1,413) = 0.00	F(1,413) = 0.01	χ[1] = 8.29**, OR = 0.43	
**Monitor**	**Total Model**	F(2,405) = 4.15*, R^2 ^= 0.02	**F(2,405) = 0.09**	**χ [2] = 4.21**	**χ [2] = 3.55**
	Child-report	F(1,405) = 6.10*, β = 0.13			
	Parent-report	F(1,405) = 0.35			
**Health Literacy**	**Total Model**	F(2,403) = 6.98***, R^2 ^= 0.03	**F(2,403) = 0.41**	**χ [2] = 1.35**	**χ [2] = 0.51**
	Child HL	F(1,403) = 11.16***, β = 0.17			
	Parent HL	F(1,403) = 0.26			

Notes: DV = dependent variable, IV = Independent variable in the model being assessed. OR = Odds ratio. ^a^ = β for Gender is based on female = 1, male- = 2, with a positive beta indicating higher levels of the construct for males. ^b^ = effects of Gender and Residence were not estimable for Smoking, because all participants who reported smoking were male, and from non-urban areas. ^c^ = Norms are scored so that higher scores represent higher perceived normative levels for the behavior; i.e. for Healthy Diet and Physical Activity higher norms represent higher levels of healthy behavior, whereas for Drinking and Smoking higher norms represent higher levels of unhealthy behavior. ‘Total Model’ refers to the simultaneous effects of the subscales of the IV (e.g. for Health Literacy: Child Health Literacy, and Parent Health Literacy), which controls for confounding across the subscales. For the AFHC and IPAQ, General Linear Model analyses were used, which produce an F statistic, with F(a, b) indicating ‘a’ numerator degrees of freedom, and ‘b’ denominator degrees of freedom; for Drinking and Smoking, ordinal logistic regression was used which produces a chi-square statistic.

Norms also had a significant overall effect on the IPAQ Physical Activity, F(4,413) = 9.04, *p* < .0001, R^2 ^= 0.08. Within this model, effects of norms regarding physical activity and drinking of alcohol were significant, F(1,413) = 19.27, *p* < .0001, *β* = 0.22 and F(1,413) = 10.82, *p* < .01, *β* = 0.20 (respectively), indicating that the more adolescents believed that being physically active and drinking alcohol was normative, the higher their reported of levels of physical activity. Because females had reported significantly lower levels of physical activity than males, we tested whether females and males differed for their norms regarding physical activity. The effect was significant, F(1,429) = 11.03, *p* < .001, R^2 ^= .03, with females about .31 of a standard deviation lower than males. To determine if this difference in norms was related to the gender difference in self-reported physical activity, we tested whether including norms in the model reduced gender differences in self-reported physical activity. Using the Hayes SAS PROCESS macro (Hayes, 2013), we found that inclusion of perceptions of norms did significantly reduce male vs. female differences in regards to physical activity, t(416) = −2.35, *p* < .05 (for the indirect effect), indicating that norms are a potential mediator of these gender differences in physical activity.

Norms also had a significant effect on adolescent reports of drinking of alcohol, χ[4] = 24.75, *p* < .0001, R^2 ^= 0.13. Within this model, the effects of norms regarding drinking of alcohol and smoking of tobacco were significant, χ[1] = 24.25, *p* < .0001, OR = 3.62; χ[1] = 8.29, *p* < .01, OR = 0.43, respectively. Thus, the higher the perceived norm for drinking, the higher the level on the ordinal drinking scale that the adolescent reported whereas in contrast, the higher the perceived norm for smoking the lower the level the adolescent reported on the ordinal drinking scale. The overall effect of norms on adolescent reports of smoking was non-significant.

The next analyses focused on the adolescent-report and parent-report parental monitoring variables, with the two variables analyzed in the same general linear models. For Unhealthy Diet, the overall effect of the model was significant, F(2,405) = 4.15, *p* < .05, R^2^ = 0.02, indicating that parental monitoring was related to adolescents’ reports of the extent to which they engaged in healthy diet behaviors. Within this model, the child-report of parental monitoring was significant, F(1,405) = 6.10, *p* < .001, with *β* = 0.13. Thus, the more the child reported that the parent monitored their behavior, the healthier the diet the child reported. Effects of parental monitoring on the other HRB were non-significant.

The final analyses focused on adolescent and parent reports of health literacy, which were analyzed similarly to the parental monitoring variables. For Unhealthy Diet, the overall model was significant, F(2,403) = 6.98, *p* < .001, R^2 ^= 0.03, indicating that health literacy was related to levels of unhealthy Diet. Within this model, the effect of adolescent health literacy was significant, t(1) = 3.34, *p* < .001, β = 0.17, indicating that the higher the level of adolescent health literacy, the lower the levels of reported unhealthy diet. The effects of health literacy on the other three HRBs were non-significant.

## Discussion

There have been relatively few studies investigating the ‘Big Four’ HRBs and their co-occurrence in LMIC, and the present study is the first assessing these four behaviors in Vietnam. Understanding their co-occurrence and common risk factors is important because these HRBs impact on health in overlapping processes with cumulative effects, which means that prevention programs may be most efficient when considering the HRBs together (Alamian & Paradis, 2012). For instance, as discussed below, our results indicated that norms for physical activity were linked to healthy diet as well as physical activity, which suggests that norms for physical activity may be a particularly important intervention target.

Overall, the prevalence of HRBs among the Vietnamese adolescents in our sample was high, with over half of the sample (59%) engaged in one or more of HRBs. This is similar to other studies in LMICs such as Brazil, Turkey, Laos and Cambodia (Brito et al., 2015; Geckil & Dündar, 2011; Sok et al., 2020; Sychareun, Thomsen, & Faxelid, 2011). In our sample, unhealthy diet (e.g. consumption of fats, sugars, fast-food, low intake of fruits, vegetables) was the most frequent (45%) HRB reported. Studies in high income countries (e.g. Korea, Italy, Australia) (Chung, Ersig, & McCarthy, 2019; Viggiano et al., 2015; Williams & Mummery, 2012) and in Western countries (Inchley et al., 2020) have similarly found that unhealthy eating behavior is a common problem among adolescents. This suggests that even in LMIC countries such as Vietnam where the fast food and similar industries are less developed and where farming remains a central part of the national identity, unhealthy diet among adolescents is a major HRB. There are several factors that may make unhealthy diet relatively common during adolescence. First, nutritional needs increase during adolescence because of the increased growth rate and changes in body composition associated with puberty, resulting in increased interest in food consumption irrespective of health (World Health Organization, 2005). In addition, factors such as a desire for independence and acceptance by peers, increased mobility, and greater time spent outside the home may result in adolescents making less healthy food choices (Lowe, Morton, & Reichelt, 2020).

The second most frequent HRB was low levels of physical activity (23%). Rates of physical inactivity among youth in developing countries has been found to vary significantly across countries and geographical regions, even when using the same measure (the IPAQ), which may be due in part to the fact there is no generally agreed upon cut-off for IPAQ physical activity vs inactivity. More generally, there is no inherent cut-point for defining ‘unhealthy’ levels of physical inactivity (as well as unhealthy diet), whereas alcohol and tobacco use can, and do, have a simple but logical cutoff for adolescents: any use is unhealthy. Our results were similar to other studies which defined physical inactivity as (a) less than 30 min of moderate to intense activity 5 days a week, and / or (b) less than 20 min of vigorous activity 3 days a week. For example, Pengpid et al. (2016) and Sok et al. (2020) in South Asia and China, and South East Asia reported rates of physical inactivity of 27% and 22%, respectively. With the same cut-off for physical inactivity, the crude worldwide prevalence of physical inactivity among adolescents using the above criterion was 21% (Dumith et al., 2011). However, the prevalence of physical inactivity in our study is much lower than that reported in other studies in the region that used a threshold of ‘physical inactivity’ of less than 60 min of moderate to vigorous-intensity physical activity per day at least 5 days per week (e.g. Peltzer and Pengpid (2016)). One limitation of research on physical inactivity, as well as for research on unhealthy diet, is that there has been little study of the validity of cut-points for measures such as the IPAQ. Further research to establish clear criteria for unhealthy levels of physical activity and for unhealth dietary behaviors will be important in order to provide meaningful estimates of the rates of these health risk behaviors. This will be important to help determine how the prevalence of low physical activity differs across countries, and to identify potential socio-cultural factors underlying these differences.

One of the most important findings in regards to this HRB was that females showed significantly higher rates of physical inactivity than males, which is in line with global findings (Guthold, Stevens, Riley, & Bull, 2020). Several possible explanations have been suggested. First, biological factors may contribute to sex differences in physical activity, with girls’ earlier physical and social maturation potentially linked to developmentally early reductions in adolescence-related increases in levels of physical activity (Sember, Jurak, Kovač, Đurić, & Starc, 2020). Second, females typically receive less peer support for engaging in physical activity than males do (Edwardson, Gorely, Pearson, & Atkin, 2013), which was supported by our finding that perceived social norms for physical activity partly explained the lower levels of physical activity for females in our study. Finally, support for sports participation and similar physical activities also appears to be weaker for girls than for boys (Telford, Telford, Olive, Cochrane, & Davey, 2016).

The prevalence of alcohol consumption in the past 30 days by our high school adolescents was relatively high, about 13%. This r may reflect the fact that in some parts of Vietnamese society, a ‘drinking culture’ is prevalent (Lincoln, 2016). For instance, consumption of alcoholic beverages – beer, rice wine, liquor, etc. –is an integral part of many social occasions or ceremonies, such as traditional holidays (e.g. Lunar New Year), and for rites of passage such as weddings, funerals, etc. Alcohol also is often a part of business transactions and everyday sociality which provides role models for adolescent drinking. Consequently, adolescent drinking of alcohol can be an acceptable social behavior, particularly within the family, and can be encouraged even from an early age, potentially leading to harmful long-term drinking patterns (Thoa, Hoang, Nguyen, Pham, & Plant, 2013). This possibility is supported by the finding that perceived norms for drinking were relatively strongly related (β = 0.31) to reported alcohol drinking. This suggests that it may be particularly useful for anti-adolescent drinking campaigns to use key opinion leaders or other individuals respected by adolescents in Vietnam.

One positive finding in our results was that only a very small proportion of the adolescents reported tobacco smoking, with 1% reporting having smoked in the past 30 days This rate is lower than reported in some previous studies in Vietnam, with some other studies reporting 9% to 18% of youth experimenting with smoking and 3% to 5% being current smokers (Joung & Chung, 2019; Page, Thanh Huong, Chi, & Truong Quang, 2010). There are several possible explanations for this difference. First, it is possible that social desirability factors lowered the reported rate of smoking in our sample, although why that would influence our study differentially from prior studies reporting higher rates is unclear. Nor is it evident why it would differentially affect tobacco smoking vs. alcohol drinking. Second, it is possible that the lower smoking rate in our study may be due to the participants’ age range. Prior studies in Vietnam found that the higher the age range (17 - 19 years old, as compared to 15–17 years old, and 13–15 years old), the higher smoking rate (Nguyen et al., 2012; Thoa et al., 2013). Our study used a relatively younger sample, with a mean age of 15.6 years of age, which may be one reason why our rate was lower. However, smoking prevalence has been declining in Vietnam over the past ten years or so, in response to a national anti-tobacco campaign (Fuchs Tarlovsky & Gonzalez Icaza, 2019). Ultimately, however, given its very low rates, analyses involving smoking must be interpreted cautiously, because of the consequent very low statistical power.

The proportion of adolescents engaged in two or more HRBs (approximately 20%) was similar to that found in other LMIC in the region such as Cambodia (Sok et al., 2020), but somewhat lower than found in some other LMIC such as Brazil (Brito et al., 2015). The present study’s somewhat lower rates of co-occurrence may be due at least in part to differences in the overall rates between the present study and research such as that in Brazil (Brito et al., 2015), since co-occurrence will be strongly influenced by individual rates. In any case, adolescents with two or more HRB may be a particularly important group requiring special attention since future health problems can be caused by a set of aggregated risks behaviors, such as throat cancer, which can be explained by the simultaneous occurrence of two unhealthy habits (smoking and alcohol consumption) (World Health Organization, 2009).

With regards to specific areas of overlap, the most frequent co-occurring HRBs were unhealthy diet and low levels of physical activity, which likely reflects in part the logic of these two behaviors; i.e. individuals interested in supporting their health may target two major, obvious areas: diet and physical activity. The second most frequent co-occurring HRBs were physical inactivity and drinking, which were negatively linked; i.e. the more physical activity that the adolescent reported (i.e. in a healthy direction), the more drinking behavior the adolescent reported (i.e. in an unhealthy direction). Results from other studies have been somewhat inconsistent in regards to this finding. Some studies have found minimal relations (Niedermeier, Frühauf, Kopp-Wilfling, Rumpold, & Kopp, 2018), negative relations (Badicu, Zamani Sani, & Fathirezaie, 2020), or positive relations (Leasure, Neighbors, Henderson, & Young, 2015) between physical activity and alcohol consumption. Leasure et al. (2015), for instance, found that similar to our results moderate drinkers were more likely to adopt physically active lifestyles. This may be the result of drinking and physical activity being seen as indicators of masculinity in Vietnam, as well as in many other countries (Lincoln, 2016), which if further validated may provide an important intervention target.

In regards to HRB risk factors, the most consistent factor was adolescents’ perceptions of norms regarding HRB and related healthy behaviors. To our knowledge, this is the first study in this area in Vietnam, with our findings supporting the social norms theory of health behaviors (Perkins, 2003). Perceptions of peer and parents adopting or supporting health-related behaviors (‘descriptive norms’) have been found to be among the most consistent, strongest, and proximal predictors of adolescents’ smoking (Eisenberg, Toumbourou, Catalano, & Hemphill, 2014), drinking (Leung, Toumbourou, & Hemphill, 2014), diet (Stok, de Vet, de Ridder, & de Wit, 2016), and physical activity (Draper, Grobler, Micklesfield, & Norris, 2015). Culturally, Vietnam is widely considered to be a collectivist society with a highly group-oriented culture (Thêm, 2003). Group-oriented individuals have been found to be more likely to adhere to social norms (Lapinski & Rimal, 2005) to maintain group harmony and interdependence, which may explain the effect of norms on health risk behaviors. As would be expected, the strongest predictor for norms was the within domain predictor (e.g. norms for physical activity were the strongest predictor of physical [in]activity). There were, however, also cross-domain predictors, which suggests that the cultural desire for belonging to the community may include a general sense of ‘healthy’ (or perhaps conversely, ‘rebelliousness’) that extends beyond single HRB domains. These findings support the importance of interventions working with opinion leaders, within the peer group to establish healthy norms for their group.

One area where there was a notable difference between the present study and prior research was the effects of parental monitoring. In Western studies, parental monitoring has been found to be a strong protective factor for adolescent risk behaviors such as alcohol drinking and tobacco smoking (Hemovich, Lac, & Crano, 2011; Thompson, Roemer, & Leadbeater, 2015). In contrast, in the present study, the only HRB related to parental monitoring was adolescents’ dietary behaviors. There is limited research in Vietnam to compare to our study, with only one other study assessing relations between parental monitoring and health risk behaviors in Vietnam. Vo (2020) focused on adolescents in nine Southeast Asian countries including Vietnam. The study found that parental monitoring scores were associated with reduced risk for adolescent alcohol use, tobacco smoking, substance abuse, and risky sexual behavior. The World Health Organization measure of parental monitoring had two items, one asking whether parents knew how their children spent their spare time, and the other how much the parents understood their child’s concerns and problems. The second item thus did not directly assess parent monitoring per se but rather parents’ understanding and caring for their child, which may the actual factor linked to positive adolescent functioning. Although the Vo (2020) study provides important information regarding risk factors for Vietnamese and other Southeast Asian adolescents’ HRB, it is difficult to compare results of that study with current study results.

One possible reason that diet may have been the only area linked to parental monitoring among our sample is that in Vietnam, in addition to parents’ generally providing breakfast and dinner and hence being able to monitor and control diet, students often bring lunch from home or return home for lunch, which means that adults also have some control of what is eaten at lunch. On the other hand, in regards to physical activity, during the school year at least, adolescents spend much of their time at school so parents may have less awareness and control of their physical activity. Also, in contrast to diet and eating, parents generally are less directly involved with their adolescents’ physical activity (e.g. whereas parents generally eat meals with their children, they do not play sports with them).

Our final risk / protective factor, health literacy, also had relatively few significant relations to HRB. Although health literacy has been found to be crucial for the development health-supportive behaviors and health outcomes in adults (DeWalt & Hink, 2009), research on the association between health literacy and adolescent health-related behavior is relatively limited. In a systematic review of adolescents’ health literacy and health behaviors, Fleary, Joseph, and Pappagianopoulos (2018) summarized results of 17 studies, and found a significant relationship between health literacy and adolescents’ health behaviors. However, the definition of health literacy in these studies varied, focusing on the application of literacy and numeracy skills to health-related materials such as medicine labels and prescriptions, and the ability to navigate within healthcare system. These operationalizations of health literacy are different from our questionnaire, which measured knowledge regarding healthy eating and physical activity for health, the consequences of smoking and drinking, etc.

In our sample, the only significant effect for health literacy was the relation between adolescents’ health literacy and unhealthy eating. This may because diet can have a relatively rapid impact on health-related areas to adolescents, such as physical appearance and body shape. In contrast, effects of HRB such as smoking, drinking, and physical activity may have more delayed impact, reducing effects of adolescents’ literacy on such behaviors, as adolescence is a period of time when the individuals generally are less focused on and aware of long-term consequences of behavior, more focused on immediate effects. This process can be measured through the behavioral paradigm known as delay discounting (Steinberg et al., 2009). As a behavioral variable, delay discounting describes the extent to which an individual discounts the value of an outcome because of a delay to its occurrence. Research has shown that the discounting rates are highest during adolescence, which could explain why more delayed outcomes, such as for smoking, etc. were not related to health literacy in our sample (Reynolds & Fields, 2012).

These findings have several important policy implications. First, the results indicate that more attention on adolescent HRB from the Vietnamese governmental and NGO different sectors is warranted, given the fact that almost 60% of Vietnamese adolescents in the sample engaged in one or more HRB, with about 20% engaged in two or more HRB. As discussed below, the fact that the sample included only students is a limitation, but it also suggests a policy focus on school-based interventions could be useful. In addition, although the HRB assessed were not correlated in our sample, they did co-occur, with about one-third of students reporting HRB reporting more than one HRB. This suggests that policy initiatives might be most productive to focus simultaneously on multiple HRBs. This also suggests that promotion, prevention and intervention programs for HRBs should address multifactorial risk and protective factors, such as health literacy, social norm, parental monitoring, which should be within the specific socio-cultural and gender specific context.

Our findings should be interpreted in the context of several limitations. First, the cross-sectional design does not allow for assessment of causality or directionality between health risk behaviors and the risk factors. Future research using a longitudinal design will be necessary to assess causal relations. A longitudinal design also could assess the sequence of co-occurrence of HRB (e.g. whether unhealthy diet comes before low physical activity), which could be useful for structuring prevention programs. Second, reliance on self-report of HRB may have resulted in social desirability or other self-report biases, particularly for sensitive information such as smoking and drinking. It will be useful for future studies to adopt objective measurement and devices, such as armband sensors to record physical activity, etc. Finally, broad generalization of results must be made with caution, as the sample size of the study was relatively small in relation to the number of analyses conducted, and only adolescents attending public schools in five provinces in Vietnam were included in the sample. Results might differ in samples of adolescents not enrolled in the educational system.

## References

[CIT0001] Alamian, A., & Paradis, G. (2012). Individual and social determinants of multiple chronic disease behavioral risk factors among youth. *BMC Public Health*, *12*(1), 1–15. 22439966 10.1186/1471-2458-12-224 PMC3331803

[CIT0002] Badicu, G., Zamani Sani, S. H., & Fathirezaie, Z. (2020). Predicting tobacco and alcohol consumption based on physical activity level and demographic characteristics in Romanian students. *Children and Youth Services Review*, *7*(7), 71. 10.3390/children7070071 PMC7401875 32630729

[CIT0003] Ball, K., Jeffery, R. W., Abbott, G., McNaughton, S. A., & Crawford, D. (2010). Is healthy behavior contagious: Associations of social norms with physical activity and healthy eating. *International Journal of Behavioral Nutrition and Physical Activity*, *7*(1), 86. doi:10.1186/1479-5868-7-86 PMC3018448 21138550

[CIT0004] Brito, A. L. D. S., Hardman, C. M., & Barros, M. V. G. D. (2015). Prevalence and factors associated with the co-occurrence of health risk behaviors in adolescents. *Revista Paulista de Pediatria*, *33*(4), 423–430. 26298656 10.1016/j.rpped.2015.02.002 PMC4685562

[CIT0001a] Byrne, B. M. (2016). Adaptation of assessment scales in cross-national research: Issues, guidelines, and caveats. *International Perspectives in Psychology: Research, Practice, Consultation*, *5*(1), 51–65. 10.1037/ipp0000042

[CIT0005] Campbell, R., Wright, C., Hickman, M., Kipping, R. R., Smith, M., Pouliou, T., & Heron, J. (2020). Multiple risk behaviour in adolescence is associated with substantial adverse health and social outcomes in early adulthood: Findings from a prospective birth cohort study. *Preventive Medicine: An International Journal Devoted to Practice and Theory*, *139*(7), 106157. 10.1016/j.ypmed.2020.106157 PMC7378566 32473267

[CIT0006] Chung, S. J., Ersig, A. L., & McCarthy, A. M. (2019). Diet and physical activity of Korean female adolescents in their peer networks. *. Journal of Nursing Scholarship*, *51*(2), 147–156. 30548904 10.1111/jnu.12453

[CIT0007] Currie, C., Gabhainn, S. N., & Godeau, E. (2009). The health behaviour in school-aged children: WHO collaborative cross-national (HBSC) study: Origins, concept, history and development 1982–2008. *International Journal of Public Health*, *54*(2), 131–139. 10.1007/s00038-009-5404-x 19639260

[CIT0008] Dang, H. M., Nguyen, H., & Weiss, B. (2017). Incremental validity of the Child Behavior Checklist (CBCL) and the Strengths and Difficulties Questionnaire (SDQ) in Vietnam. *Asian Journal of Psychiatry*, *29*, 96–100. 29061439 10.1016/j.ajp.2017.04.023 PMC5679093

[CIT0009] Dang, H. M., Weiss, B., Pollack, A., & Nguyen, M. C. (2012). Adaptation of the Wechsler intelligence scale for children-IV (WISC-IV) for Vietnam. *Psychological Studies*, *56*(4), 387–392. 23833330 10.1007/s12646-011-0099-5 PMC3702178

[CIT0010] DeWalt, D. A., & Hink, A. (2009). Literacy and child health outcomes: A systematic review of the literature. *Pediatrics*, *124*(Supplement 3), S265–S274. 19861480 10.1542/peds.2009-1162B

[CIT0011] DiClemente, R. J., Hansen, W. B., & Ponton, L. E. (2013). *Handbook of adolescent health risk behavior*. Berlin: Springer Science & Business Media.

[CIT0012] DiClemente, R. J., Santelli, J. S., & Crosby, R. A. E. (Eds.), (2009). *Adolescent health: Understanding and preventing risk behaviors*. San Francisco, CA: John Wiley & Sons.

[CIT0013] Djoussé, L., Driver, J. A., & Gaziano, J. M. (2009). Relation between modifiable lifestyle factors and lifetime risk of heart failure. *JAMA*, *302*, 394–400. 19622818 10.1001/jama.2009.1062 PMC2742484

[CIT0014] Draper, C. E., Grobler, L., Micklesfield, L. K., & Norris, S. A. (2015). Impact of social norms and social support on diet, physical activity and sedentary behaviour of adolescents: A scoping review. *Child: Care, Health and Development*, *41*(5), 654–667. 10.1111/cch.12241 25809525

[CIT0002a] Dumith, S. C., Muniz, L. C., Tassitano, R. M., Hallal, P. C., & Menezes, A. M. (2012). Clustering of risk factors for chronic diseases among adolescents from Southern Brazil. *Preventive medicine*, *54*(6), 393–396. 22484392 10.1016/j.ypmed.2012.03.014 PMC4210640

[CIT0015] Dumith, S. C., Hallal, P. C., Reis, R. S., & Kohl III, H. W. (2011). Worldwide prevalence of physical inactivity and its association with human development index in 76 countries. *Preventive Medicine*, *53*(1–2), 24–28. 21371494 10.1016/j.ypmed.2011.02.017

[CIT0016] Edwardson, C. L., Gorely, T., Pearson, N., & Atkin, A. (2013). Sources of activity-related social support and adolescents’ objectively measured after-school and weekend physical activity: Gender and age differences. *Journal of Physical Activity and Health*, *10*(8), 1153–1158. 23223792 10.1123/jpah.10.8.1153

[CIT0017] Eisenberg, M. E., Toumbourou, J. W., Catalano, R. F., & Hemphill, S. A. (2014). Social norms in the development of adolescent substance use: A longitudinal analysis of the International Youth Development Study. *Journal of Youth and Adolescence*, *43*(9), 1486–1497. 24633850 10.1007/s10964-014-0111-1 PMC4130778

[CIT0018] Fleary, S. A., Joseph, P., & Pappagianopoulos, J. E. (2018). Adolescent health literacy and health behaviors: A systematic review. *Journal of Adolescence*, *62*, 116–127. 29179126 10.1016/j.adolescence.2017.11.010

[CIT0019] Fuchs Tarlovsky, A., & Gonzalez Icaza, F. (2019). *The welfare and distributional effects of increasing taxes on tobacco in Vietnam*. Hanoi: World Bank.

[CIT0020] Geckil, E., & Dündar, Ö. (2011). Turkish adolescent health risk behaviors and self-esteem. *Social Behavior and Personality: An International Journal*, *39*(2), 219–227.

[CIT0021] General Statistics Office of Vietnam. (2019). *Statistical censuses and surveys*. Hanoi: GSO. Retrieved from http://tongdieutradanso.vn/project-of-the-census.html

[CIT0022] Guthold, R., Stevens, G. A., Riley, L. M., & Bull, F. C. (2020). Global trends in insufficient physical activity among adolescents: A pooled analysis of 298 population-based surveys with 1.6 million participants. *The Lancet Child & Adolescent Health*, *4*(1), 23–35. 31761562 10.1016/S2352-4642(19)30323-2 PMC6919336

[CIT0003a] Hambleton, R. K., Merenda, P. F., & Spielberger, C. (2005). *Adapting educational and psychological tests for cross-cultural assessment*. Mahwah: Lawrence Erlbaum Associates.

[CIT0023] Hale, D. R., & Viner, R. M. (2016). The correlates and course of multiple health risk behaviour in adolescence. *BMC Public Health*, *16*(1), 1–12. 27246600 10.1186/s12889-016-3120-z PMC4888596

[CIT0024] Hayes, A. F. (2013). *Introduction to mediation, moderation, and conditional process analysis: A regression-based approach*. NYC: The Guilford Press.

[CIT0025] Hemovich, V., Lac, A., & Crano, W. D. (2011). Understanding early-onset drug and alcohol outcomes among youth: The role of family structure, social factors, and interpersonal perceptions of use. *Psychology, Health & Medicine*, *16*(3), 249–267. 10.1080/13548506.2010.532560 PMC3088114 21491334

[CIT0026] Heneghan, A., Stein, R. E., Hurlburt, M. S., Zhang, J., Rolls-Reutz, J., Kerker, B. D., & Horwitz, S. M. (2015). Health-risk behaviors in teens investigated by US child welfare agencies. *Journal of Adolescent Health*, *56*(5), 508–514. 10.1016/j.jadohealth.2015.01.007 PMC5564320 25744208

[CIT0027] Inchley, J., Currie, D., Budisavljevic, S., Torsheim, T., Jåstad, A., Cosma, A. … Samdal, O.. (2020). *Spotlight on adolescent health and well-being. Findings from the 2017/2018 Health Behaviour in School-aged Children (HBSC) survey in Europe and Canada. International report. Volume 1. Key findings*. Copenhagen: WHO Regional Office for Europe

[CIT0028] IPAQ Research Committee. (2005). Guidelines for data processing and analysis of the International Physical Activity Questionnaire (IPAQ)-short and long forms. Retrieved from http://www.ipaq.ki.se/scoring.pdf

[CIT0029] Jackson, C. A., Henderson, M., Frank, J. W., & Haw, S. J. (2012). An overview of prevention of multiple risk behaviour in adolescence and young adulthood. *Journal of Public Health*, *34*(suppl_1), i31–i40. 22363029 10.1093/pubmed/fdr113

[CIT0030] Jessor, R. (2016). *The origins and development of problem behavior theory*. New York: Springer.

[CIT0031] Jessor, R., & Turbin, M. S. (2016). Problem behavior theory and adolescent Pro-social behavior. In R. Jessor (Ed.), *The Origins and development of problem behavior Theory* (pp. 181–203). Cham: Springer.

[CIT0032] Jessor, R., Turbin, M. S., & Costa, F. M. (1998). Protective factors in adolescent health behavior. R. Jessor (Ed.), *Journal of Personality and Social Psychology*, *75*(3), 788–800. 9781412 10.1037//0022-3514.75.3.788

[CIT0033] Johnson, F., Wardle, J., & Griffith, J. (2002). The adolescent food habits checklist: Reliability and validity of a measure of healthy eating behaviour in adolescents. *European Journal of Clinical Nutrition*, *56*(7), 644. 12080404 10.1038/sj.ejcn.1601371

[CIT0034] Joung, K. H., & Chung, S. S. (2019). Factors affecting cigarette smoking among adolescents in South Korea, Vietnam, and Thailand. *Journal for Specialists in Pediatric Nursing*, *24*(4), e12267. doi:10.1111/jspn.12267 31468713

[CIT0035] Kabir, A., Miah, S., & Islam, A. (2018). Factors influencing eating behavior and dietary intake among resident students in a public university in Bangladesh: A qualitative study. *PloS one*, *13*(6), e0198801. 29920535 10.1371/journal.pone.0198801 PMC6007825

[CIT0036] Kalkan, I. (2019). The impact of nutrition literacy on the food habits among young adults in Turkey. *Nutrition Research and Practice*, *13*(4), 352–357. 31388412 10.4162/nrp.2019.13.4.352 PMC6669071

[CIT0037] Kann, L., McManus, T., Harris, W. A., Shanklin, S. L., Flint, K. H., Queen, B., & Ethier, K. A. (2018). Youth risk behavior surveillance—United States, 2017. *MMWR Surveillance Summaries*, *67*(8), 1. 10.15585/mmwr.ss6708a1 PMC6002027 29902162

[CIT0038] Lapinski, M. K., & Rimal, R. N. (2005). An explication of social norms. *Communication Theory*, *15*(2), 127–147.

[CIT0039] Leasure, J. L., Neighbors, C., Henderson, C. E., & Young, C. M. (2015). Exercise and alcohol consumption: What we know, what we need to know, and why it is important. *Frontiers in Psychiatry*, *6*, 156. 26578988 10.3389/fpsyt.2015.00156 PMC4629692

[CIT0040] Leung, R. K., Toumbourou, J. W., & Hemphill, S. A. (2014). The effect of peer influence and selection processes on adolescent alcohol use: A systematic review of longitudinal studies. *Health Psychology Review*, *8*(4), 426–457. 25211209 10.1080/17437199.2011.587961

[CIT0041] Levine, C. S., Miyamoto, Y., Markus, H. R., Rigotti, A., Boylan, J. M., Park, J., & Ryff, C. D. (2016). Culture and healthy eating: The role of independence and interdependence in the United States and Japan. *Personality and Social Psychology Bulletin*, *42*(10), 1335–1348. 27516421 10.1177/0146167216658645 PMC5023492

[CIT0042] Lincoln, M. (2016). Alcohol and drinking cultures in Vietnam: A review. *Drug and Alcohol Dependence*, *159*, 1–8. doi:10.1016/j.drugalcdep.2015.10.030 26802499 PMC4725306

[CIT0043] Lowe, C. J., Morton, J. B., & Reichelt, A. C. (2020). Adolescent obesity and dietary decision making—A brain-health perspective. *The Lancet Child & Adolescent Health*, *4*(5), 388–396. 32164832 10.1016/S2352-4642(19)30404-3

[CIT0044] Nguyen, H. T., Tang, K. H., Dibley, M. J., & Sibbritt, D. W. (2009). Factors associated with physical inactivity in adolescents in Ho Chi Minh City, Vietnam. *Medicine & Science in Sports & Exercise*, *41*(7), 1374–1383. 19516164 10.1249/MSS.0b013e31819c0dd3

[CIT0045] Nguyen, L. T., Rahman, Z., Emerson, M. R., Nguyen, M. H., & Zabin, L. S. (2012). Cigarette smoking and drinking behavior of migrant adolescents and young adults in Hanoi. *Vietnam. Journal of Adolescent Health*, *50*(3), S61–S67. 10.1016/j.jadohealth.2011.12.004 PMC4169841 22340858

[CIT0046] Nguyen, P. V. N., Hong, T. K., Hoang, T., Nguyen, D. T., & Robert, A. R. (2013). High prevalence of overweight among adolescents in Ho Chi Minh City, Vietnam. *BMC Public Health*, *13*(1), 141. doi:10.1186/1471-2458-13-141 23414441 PMC3598401

[CIT0047] Niedermeier, M., Frühauf, A., Kopp-Wilfling, P., Rumpold, G., & Kopp, M. (2018). Alcohol consumption and physical activity in Austrian college students—A cross-sectional study. *Substance use & Misuse*, *53*(10), 1581–1590. 29381116 10.1080/10826084.2017.1416406

[CIT0048] Page, R., Thanh Huong, N., Chi, H., & Truong Quang, T. (2010). Social normative beliefs about smoking Among Vietnamese adolescents. *Asia-Pacific Journal of Public Health / Asia-Pacific Academic Consortium for Public Health*, *24*, 68–81. doi:10.1177/1010539510370993 20498122

[CIT0049] Peltzer, K., & Pengpid, S. (2016). Leisure time physical inactivity and sedentary behaviour and lifestyle correlates among students aged 13–15 in the association of Southeast Asian nations (ASEAN) member states, 2007–2013. *International Journal of Environmental Research and Public Health*, *13*(2), 217. 26891312 10.3390/ijerph13020217 PMC4772237

[CIT0050] Pengpid, S., Peltzer, K., Kassean, H. K., Tsala, J. P. T., Sychareun, V., & Müller-Riemenschneider, F. (2016). Physical inactivity and associated factors among university students in 23 low-, middle-and high-income countries. *International Journal of Public Health*, *60*(5), 539–549. 10.1007/s00038-015-0680-0 25926342

[CIT0051] Perkins, H. W. (2003). The emergence and evolution of the social norms approach to substance abuse prevention. In H. W. Perkins (Ed.), *The social norms approach to preventing school and college age substance abuse* (pp. 3–17). San Francisco, CA: Jossey-Bass/Wiley.

[CIT0052] Phuong, T. B., Huong, N. T., Tien, T. Q., Chi, H. K., & Dunne, M. P. (2013). Factors associated with health risk behavior among school children in urban Vietnam. *Global Health Action*, *6*(1), 18876. doi:10.3402/gha.v6i0.18876 PMC3549466 23336622

[CIT0053] Reitsma, M. B., Fullman, N., Ng, M., Salama, J. S., Abajobir, A., Abate, K. H., … Gakidou, E. (2017). Smoking prevalence and attributable disease burden in 195 countries and territories, 1990–2015: A systematic analysis from the Global Burden of Disease Study 2015. *The Lancet*, *389*(10082), 1885–1906. 10.1016/S0140-6736(17)30819-X PMC5439023 28390697

[CIT0054] Reynolds, B., & Fields, S. (2012). Delay discounting by adolescents experimenting with cigarette smoking. *Addiction*, *107*(2), 417–424. 21906199 10.1111/j.1360-0443.2011.03644.x PMC3260343

[CIT0055] Sember, V., Jurak, G., Kovač, M., Đurić, S., & Starc, G. (2020). Decline of physical activity in early adolescence: A 3-year cohort study. *PloS one*, *15*(3), e0229305. 32160216 10.1371/journal.pone.0229305 PMC7065740

[CIT0056] Sok, S., Pal, K., Tuot, S., Yi, R., Chhoun, P., & Yi, S. (2020). Health behaviors among male and female university students in Cambodia: A cross-sectional survey. *Journal of Environmental and Public Health*, *2020*, Article ID 6740236. 10.1155/2020/6740236 PMC7086439 32256617

[CIT0057] Spring, B., Moller, A. C., & Coons, M. J. (2012). Multiple health behaviours: Overview and implications. *Journal of Public Health (Oxford, England)*, *34*(Suppl 1), i3–i10. doi:10.1093/pubmed/fdr111 PMC3284863 22363028

[CIT0058] Spybrook, J., Bloom, H., Congdon, R., Hill, C., Martinez, A., & Raudenbush, S. (2011). *Optimal Design plus empirical evidence: Documentation for the “Optimal Design” software*. William T. Grant Foundation. Retrieved from https://www.stat.cmu.edu/~brian/463-663/week14/od/od-manual-20111016-v300.pdf

[CIT0059] Steinberg, L., Graham, S., O’brien, L., Woolard, J., Cauffman, E., & Banich, M. (2009). Age differences in future orientation and delay discounting. *Child Development*, *80*(1), 28–44. 19236391 10.1111/j.1467-8624.2008.01244.x

[CIT0060] Stok, F. M., de Vet, E., de Ridder, D. T., & de Wit, J. B. (2016). The potential of peer social norms to shape food intake in adolescents and young adults: A systematic review of effects and moderators. *Health Psychology Review*, *10*(3), 326–340. 26878931 10.1080/17437199.2016.1155161

[CIT0061] Stormshak, E. A., Caruthers, A., & Dishion, T. J. (2006). Child and Family Center parent survey (unpublished measure). *Available from the Child and Family Center*, *195*.

[CIT0062] Sychareun, V., Thomsen, S., & Faxelid, E. (2011). Concurrent multiple health risk behaviors among adolescents in luangnamtha province, Lao PDR. *BMC Public Health*, *11*(1), 1–10. 21232108 10.1186/1471-2458-11-36 PMC3031220

[CIT0063] Telford, R. M., Telford, R. D., Olive, L. S., Cochrane, T., & Davey, R. (2016). Why are girls less physically active than boys? Findings from the LOOK longitudinal study. *PloS one*, *11*(3), e0150041. 26960199 10.1371/journal.pone.0150041 PMC4784873

[CIT0064] Te Morenga, L., & Montez, J. M. (2017). Health effects of saturated and trans-fatty acid intake in children and adolescents: Systematic review and meta-analysis. *PloS one*, *12*(11), e0186672–e0186672. doi:10.1371/journal.pone.0186672 29149184 PMC5693282

[CIT0065] Thêm, T. N. (2003). *Recherche sur l’identité de la culture vietnamienne*. Hanoi: Editions Thế Giới.

[CIT0066] Thoa, L. T. K., Hoang, D. H., Nguyen, D. V., Pham, H. T., & Plant, M. A. (2013). Alcohol Use, risk taking, Leisure activities and health Care Use Among young people in northern Vietnam. *Central Asian Journal of Global Health*, *2*(2), doi:10.5195/cajgh.2013.10 PMC5927737 29755877

[CIT0067] Thompson, K., Roemer, A., & Leadbeater, B. (2015). Impulsive personality, parental monitoring, and alcohol outcomes from adolescence through you adulthood. *Journal of Adolescent Health*, *57*(3), 320–326. 10.1016/j.jadohealth.2015.05.005 26143959

[CIT0068] UN General Assembly. (2015). *Transforming our world: The 2030 agenda for Sustainable development*. New York, NY: UN General Assembly.

[CIT0069] US.CDC. (2015). *National Youth Tobacco survey*. Atlanta. Retrieved from https://www.cdc.gov/tobacco/data_statistics/surveys/nyts/index.htm

[CIT0070] Vandenbroucke, J. P., Von Elm, E., Altman, D. G., Gøtzsche, P. C., Mulrow, C. D., Pocock, S. J., … Initiative, S. (2007). Strengthening the reporting of observational studies in epidemiology (STROBE): explanation and elaboration. *PLoS Medicine*, *4*(10), e297. 17941715 10.1371/journal.pmed.0040297 PMC2020496

[CIT0071] Viggiano, A., Viggiano, E., Di Costanzo, A., Viggiano, A., Andreozzi, E., Romano, V., & Amaro, S. (2015). Kaledo, a board game for nutrition education of children and adolescents at school: Cluster randomized controlled trial of healthy lifestyle promotion. *European Journal of Pediatrics*, *174*(2), 217–228. 25048788 10.1007/s00431-014-2381-8

[CIT0072] Vo, T. (2020). Parental monitoring and adolescent health risk behaviors: A comparative analysis of nine Southeast Asian countries. *Research Square*. doi:10.21203/rs.3.rs-23553/v1

[CIT0073] Weinstein, N. (2004). *Human motivation and interpersonal relationships: Theory, research, and applications; human motivation and interpersonal relationships*. New York City: Springer Science + Business Media.

[CIT0074] Weiss, B., Nguyen, T., Trung, L., Ngo, V. K., & Lau, A. (2019). Tobacco smoking and antisocial deviance among Vietnamese, Vietnamese-American, and European-American adolescents. *Journal of Abnormal Child Psychology*, *47*(1), 59–69. doi:10.1007/s10802-018-0416-8 29564575 PMC6150857

[CIT0075] Williams, S. L., & Mummery, W. K. (2012). Associations between adolescent nutrition behaviours and adolescent and parent characteristics. *Nutrition & Dietetics*, *69*(2), 95–101.

[CIT0076] World Health Organization. (2003). *The Global Strategy on diet, physical activity and health*. Geneva: WHO. Retrieved from www.who.int/dietphysicalactivity/media/en/gsfs_general.pdf

[CIT0077] World Health Organization. (2005). *Nutrition in adolescence: Issues and challenges for the health sector: Issues in adolescent health and development*. Geneva: WHO.

[CIT0078] World Health Organization. (2009). *Global health risks: Mortality and burden of disease attributable to selected major risks*. Geneva: WHO.

[CIT0079] World Health Organization. (2015). *WHO global report on trends in prevalence of tobacco smoking*. Geneva: WHO.

[CIT0080] World Health Orgniazation. (2021). Noncommunicable diseases [Factsheet]. WHO. Retrieved from https://www.who.int/news-room/fact-sheets/detail/noncommunicable-diseases

[CIT0081] Xu, T., Tomokawa, S., Gregorio, Jr., E. R., Mannava, P., Nagai, M., & Sobel, H. (2020). School-based interventions to promote adolescent health: A systematic review in low-and middle-income countries of WHO Western Pacific region. *PloS one*, *15*(3), e0230046. 32134985 10.1371/journal.pone.0230046 PMC7058297

